# Musculoskeletal pain and effort-reward imbalance- a systematic review

**DOI:** 10.1186/1471-2458-14-37

**Published:** 2014-01-15

**Authors:** Peter Koch, Anja Schablon, Ute Latza, Albert Nienhaus

**Affiliations:** 1 Centre of Excellence for Epidemiology and Health Services Research for Healthcare Professionals (CVcare), University Medical Center Hamburg-Eppendorf, Martinistraße 52, Hamburg 20246, Germany; 2 Unit Prevention of Work-Related Disorders, Division Work and Health, Federal Institute for Occupational Safety and Health, Nöldnerstraße 40, Berlin 10317, Germany; 3 Health Protection Division (FBG), Institution for Statutory Accident Insurance and Prevention in the Health and Welfare Services (BGW), Pappelallee 33, Hamburg 22089, Germany

**Keywords:** Effort-reward imbalance, Musculoskeletal pain, Psychosocial work stress, Review

## Abstract

**Background:**

Musculoskeletal pain may be triggered by physical strains and psychosocial risk factors. The effort-reward imbalance model (ERI model) is a stress model which measures psychosocial factors in the working world. The question is whether workers with an effort-reward imbalance report musculoskeletal pain more frequently than those with no effort-reward imbalance. A systematic review using a best evidence synthesis approach was conducted to answer this question.

**Methods:**

A literature search was conducted for the period from 1996 to 2012, using three databases (Pubmed, Embase and PsycINFO). The research criteria related to psychosocial, work-related stress as per the ERI model and to musculoskeletal pain. A quality score was developed using various quality criteria to assess the standard of the studies. The level of evidence was graded as in (Am J Ind Med 39:180–193, 2001).

**Results:**

After applying the inclusion criteria, a total of 19 studies were included in the review: 15 cross-sectional studies, three prospective studies and one case–control study. 74% of all studies exhibited good methodological quality, 53% collected data using the original ERI questionnaire, and in 42% of the studies, there was adequate control for physical working conditions. Furthermore, different cut-off points were used to classify exposed and non-exposed individuals. On the basis of 13 studies with a positive, statistically significant association, a moderate level of evidence was inferred for the association between *effort*-*reward imbalance* and musculoskeletal pain. The evidence for a role of *over*-*commitment* and for *its interaction with effort*-*reward imbalance* was rated as inconclusive - on the basis of eight and five studies, respectively.

**Conclusions:**

On the basis of the available evidence, no reliable conclusion may be drawn about any association between the psychosocial factors ascertained using the ERI model and musculoskeletal pain. Before a reliable statement can be made on the association between ERI and musculoskeletal pain, additional longitudinal studies must be performed - with a standardised method for recording and classifying exposure, as well as control of physical confounders. Appropriate preventive measures can then be specified.

## Background

Work-related musculoskeletal disorders (MSD) are a widespread health problem in the EU Member States. MSDs represent 61% of all work-related disorders [1]. In Germany in 2010, musculoskeletal disorders reduced the country’s net output by €16 billion [2]. In the United States, healthcare expenditure attributable only to back pain reached about $26.3 billion [3].

Experts in occupational medicine consider that many factors can influence or trigger work-related musculoskeletal disorders [4]. In addition to biomechanical factors, psychosocial factors may play a role. If these risk factors could be identified, it might be possible to develop strategies to prevent musculoskeletal disorders in the workplace.

The empirical association between work-related musculoskeletal pain and psychosocial factors is inconsistent [5,6]. Psychosocial work-related stress is evaluated using a large number of different measurement concepts [5,7-9]. In addition to simple quantification methods with no theoretical basis, theory-based work-related stress models are used to ascertain psychosocial factors. Models of work-related stress have the advantage of utilising verified, standardised ascertainment tools. The study results can then be generalised and preventive measures may be developed.

There are indications that different models of work-related stress measure different psychosocial factors. In studies which record psychosocial factors using both the job demand-control model (JDC model) [10] and the effort-reward imbalance model (ERI model) [11], independent effects can be observed in connection with chronic heart diseases and depression [12,13].

Siegrist’s ERI model (1996) is based on the assumption that there should ideally be a reciprocal relationship between the work done and socially defined rewards. The employee’s health is viewed in relation to the work they do and the rewards they receive (salary, recognition, job security and promotion prospects). If there is an imbalance consisting of high performance and low rewards, Siegrist regards this as a stressful situation, which increases the risk of stress-related disorders if it persists for some time (ERI hypothesis).

One of the distinguishing features of the ERI model is the inclusion of a personal coping strategy in connection with high work-related demands: over-commitment is a motivational pattern which generates excessive commitment in conjunction with expectations of high rewards. According to Siegrist, employees with this personal characteristic are also at increased risk of developing stress-related disorders (OVC hypothesis). Siegrist concludes that workers with intrinsic over-commitment who also experience an effort-reward imbalance are at the greatest risk of becoming ill (ERI*OVC hypothesis). While the JDC model focusses only on situational patterns, the ERI model also pays attention to personal characteristics.

Furthermore, in contrast to the JDC model, the ERI model records the impact of the global economy on labour markets. Economic trends are taken into account, using the reward components of job security, salary and promotion prospects. Therefore the ERI model can be adapted to psychosocial work stress in this day and age.

By using this method and taking personal aspects into account, the ERI model is capable of mapping a range of contemporary potentially stressful situations.

The last review investigating the association between psychosocial factors captured by the ERI model and musculoskeletal pain was published in 2005 [14]. Although there have been several recent reviews and meta-analyses on the association between psychosocial factors and musculoskeletal pain since 2011, none has recorded effort-reward imbalances [7,8,15]. A systematic review was therefore conducted using a best evidence synthesis approach.

The question is whether there is an association between the psychosocial factors ascertained using the ERI model and job-related musculoskeletal pain.

The systematic review examined the following research questions:

1) Do workers with an effort-reward imbalance report musculoskeletal disorders more frequently than workers with no effort-reward imbalance (ERI hypothesis)?

2) Do over-committed workers report musculoskeletal disorders more frequently than workers who are not over-committed (OVC hypothesis)?

3) Do workers with an effort-reward imbalance and over-commitment report musculoskeletal disorders more frequently than workers with only an effort-reward imbalance and/or workers who are only over-committed (ERI*OVC hypothesis)?

## Methods

### Search and selection strategy

The search was carried out in the Pubmed, PsycINFO and Embase databases. A secondary search was completed in the reference lists of the articles included and additional reviews [5,6,14,16-19].

The query was completed in PubMed using the following search syntax:

“effort reward imbalance AND (musculoskeletal* OR shoulder pain OR neck pain OR back pain OR upper extremity pain OR lower extremity pain OR upper limb pain OR lower limb pain OR hip pain)”. The last date on which the search was carried out was 29 August 2012. Duplicates were eliminated following an amended search in the other databases.

The following inclusion criteria were defined for the selection of studies:

1. The study population is a group of workers from a specific setting or a group of workers selected from the general population.

2. ERI is examined as a risk factor for musculoskeletal pain.

3. The outcome is a musculoskeletal disorder, regardless of its localisation.

4. The study is designed as a cross-sectional, case–control or cohort study.

5. The primary publications are articles from peer-reviewed specialist journals published in English, French or German during the period between 1996 and 2012.

Titles and abstracts were screened in line with the inclusion criteria in step one, and then the full text was checked in step two. Screening was performed independently by two reviewers.

### Quality criteria and evidence categories

To take into account the risk of bias in the individual studies, quality criteria were used to assess the studies’ methodological quality.

Table 1 shows the quality criteria in five categories: study objective, study population, exposure, outcome and analysis. The items were combined differently to allow for the three different study designs. The criteria were adapted from Ariëns et al. [18] and thematically modified as appropriate.

**Table 1:** Quality assessment criteria

	**Application of the criteria to specific study designs**	**Definition of each criterion**
**Study objective**	All	A specific objective has been clearly formulated.
**Study population**	All	The response rate is at least 80%.
	Prospective	The response rate is at least 80% after at least one year or the non-responders are not selective.
	Case–control	Cases and controls originate from the same population and there is a clear definition of cases and controls.
**Exposure**	All	The original ERI questionnaire was used, including the ascertainment of over-commitment.
	All	Data on physical work-related stress was collected and taken into account in the analysis.
**Outcome**	Case–control	Prospective enrolment was used (identification of new cases and selection of controls occurs at the same time).
	Prospective	Follow-up completed at least one year later.
**Analysis**	All	Risk predictors including confidence intervals or p-values were calculated and confounding was controlled for.

The quality of each item was assessed by two reviewers independently using the grading positive (+), negative (−) or unclear (?). If an item was rated differently, the two reviewers reached a consensus. The inter-rater reliability was moderate (Cohen’s kappa: 0.41). The positive items were added for each study to give a total score. Cross-sectional studies were deemed methodologically good if they scored more than three points (maximum: five points). Case–control studies and prospective cohort studies had to score a minimum of four points to be graded as good (maximum: seven points).

For observational studies, the level of evidence for the association between psychosocial factors defined in the ERI model and musculoskeletal pain was classified as follows (Table 2) [18]:

**Table 2:** **Evidence categories for observational studies** - **based on Ariëns et al**. [18]

**Level**	**Description**
1	*Strong evidence*: Consistent findings in multiple high-score cohort and/or case- control studies.
2	*Moderate evidence*: Consistent findings in multiple cohort or case–control studies, of which only one study was of high quality.
3	*Some evidence*: Findings of one cohort or case–control study, or consistent findings in multiple cross-sectional studies, of which a least one study was of high quality.
4	*Inconclusive evidence*: All other cases (i.e. consistent findings in multiple low-quality cross-sectional studies, or inconsistent findings in multiple studies).
	Moreover, inconclusive evidence was defined as findings of only one cross-sectional study, irrespective of the quality of the study.

Results are classified as *consistent* if at least 75% of the study results were unambiguous. *Multiple* results were defined as being derived from at least *three* studies.

As a number of studies yielded several partial results, these were combined to give a main result: if at least half of the partial results were statistically significant in relation to the hypotheses, a significant statistical effect was documented for the association within the study.

By combining the study results with a quality assessment, a level of evidence was allocated to 1) the ERI hypothesis, 2) the OVC hypothesis and 3) the ERI*OVC hypothesis.

## Results

**Figure 1:**
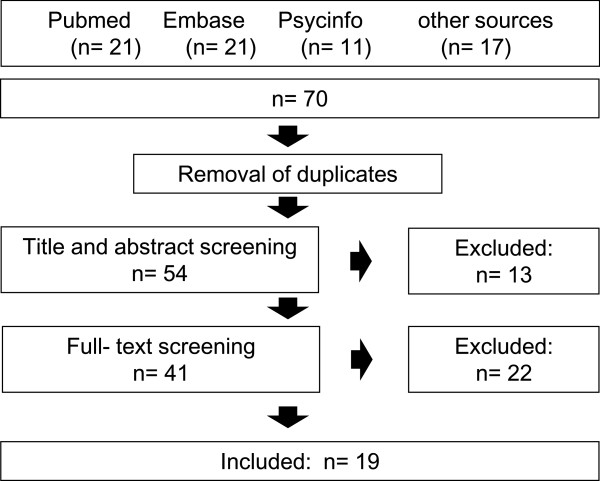
Flow chart of the inclusion process.

Seventy studies were identified in total. After eliminating duplicates and applying the inclusion criteria, 19 studies were included (Figure 1). A cross-sectional study by van Vegchel et al. [20] technically fulfilled the inclusion criteria, but was not included. This study was a further analysis of a population which had already been covered in the present review through another publication by the same authors [21]. Furthermore, this study utilised new categorisations of the model dimension of *performance* which did not originally form part of the ERI model.

Of the studies included, 15 were cross-sectional [21-35], three were prospective cohort studies [36-38] and one was a case–control study [39]. One of the prospective cohort studies [38] was a secondary analysis of an RCT examining an ergonomic intervention and musculoskeletal disorders in call centre workers [40]. Of the cross-sectional studies, three tested the psychometric properties of the translated ERI questionnaire with satisfactory results [22-24].

The following exclusion reasons were used during screening:

● The ERI questionnaire was not utilised

● No associations for effort-reward imbalance and musculoskeletal pain were measured

● Lack of data on the ascertainment of effort-reward imbalance

● Combination of different outcomes (e.g. musculoskeletal pain and psychological disorders)

● Publication in Chinese

### Description of the studies

The groups of workers examined in the included studies came from different industries. Five studies examined populations in care facilities [21,23,25,26,39] and four studies researched the association between ERI and musculoskeletal pain in employees of passenger transport companies [27-29,36], although the study by Dragano et al. [28] was a reanalysis of Joksimovic et al. [27]. This study was still included because – unlike the paper by Joksimovic et al. – it considered chronification of musculoskeletal pain. In this context, individuals were considered to be suffering from a disorder if they had experienced pain in the previous seven days - in addition to twelve-month prevalence. Lau [22] and Lapointe [37] studied public administration employees. Two studies examined random samples from surveys of the working population [30,31].

Vineyard workers [32], dental technicians [24], call centre operatives [38], cleaners [33], officers from special police units [34] and bank/insurance staff have also been studied [35].

Seven studies examined associations between ERI and other outcomes: self-related poor health and psychological distress [22,31], work-related burnout [22], poor general health, lack of vitality, poor psychological well-being [23], gastrointestinal complaints [23,29], cardiovascular complaints [23], psychosomatic health complaints and exhaustion [21,30], fatigue, sleep disturbances, common cold, nausea and dizziness [29], arterial hypertension [35], job dissatisfaction [30].

In addition to the psychosocial factors ascertained using the ERI model, ten studies [25-27,30-33,36,37,39] analysed other psychosocial factors. These were based on the demand-control model [10], the Copenhagen Psychosocial Questionnaire (COPSOQ) [41] and the Nursing Work Index – Extended Organisation Questionnaire (NWI-EO Questionnaire) [42].

### Quality criteria

All but one of the studies [39] (95%) had a clearly defined objective (no table). Only two studies (11%) achieved a response rate of at least 80% [26,36]. All of the prospective cohort studies reported a response rate of at least 80% at the time of the follow-up. The only case–control study did not provide a clear definition of cases and controls. Half of the studies which were included (53%) ascertained psychosocial factors using the original ERI questionnaire [22-25,27,28,32,34,35,37]. The other studies used proxy ERI questionnaires or did not ascertain the intrinsic component of over-commitment. A proxy ERI questionnaire refers to when the dimensions of the ERI model were used and the ERI score calculated, but the items of the dimensions were adapted to a specific work situation. A minority of studies (42%) recorded physical factors and controlled for these in the multivariate analysis [25,26,32,33,36-39]. All but one study recorded physical factors by means of self-reported data. The items assessed ergonomic, postural aspects as well as information on physical workload, work intensification and physical factors at home. Gillen et al [39] assessed physical factors with the help of an ergonomic observation. Two studies [28,34] which only recorded physical factors with the aid of one question (“My job is physically demanding”) did not fulfil this criterion because they were too vague. Other studies neglected to describe their approach in the methodological section but nevertheless presented results on physical factors; these also failed to meet this quality criterion [29,35]. The quality criterion *prospective enrolment* was fulfilled by the only case–control study [39]. All of the prospective cohort studies recorded data on the outcome after at least one year. The majority (89%) of the studies presented risk predictors with confidence intervals and controlled for confounding. Using the point system for quality criteria, 14 of the 19 studies were deemed to be of good quality (74%).

### Results of the individual studies

According to Siegrist [43], the ratio score of effort (numerator) and reward (denominator) is calculated as follows: ∑ effort/∑ reward * correction factor (correction factor for the difference in the numbers of items of the two scales).

Twelve studies used a dichotomous ERI variable (ERI ratio score values > 1 vs. values ≤ 1) in their calculations. Three of these studies included additional ERI variables in the statistical models, using tertiles, quartiles and the continuous ERI ratio score [25,33,38] (Table 3).

**Table 3:** Results by population

***Source***	***Population***	***N***	***Design***	***P/I of musculoskeletal pain***	***ERI ratio score* > *1***	***Calculation of ERI***	***Pain localisation***	***Analysis in subgroups***
**Herin et al., 2011 **[25]	Healthcare workers	2,194	**C**-**S**	P: 31%	10.7%	> 1, tertiles, continuous	Upper extremities	
**van Vegchel et al., 2002 **[21]		167	**C**-**S**	P: 13%	-	Tertiles	Upper/lower extremities, neck, shoulders, back	
**Weyers, 2006 **[23]		367	**C**-**S**	-	-	Tertiles	General MSDs	
**Simon et al., 2008 **[26]		21,516	**C**-**S**	P: 38–48%	-	Tertiles	Neck, lower back	Three facility types
**Gillen et al., 2007 **[39]		664	**C**-**C**	NA	-	Continuous	Upper/lower extremities, neck, back	
**Joksimovic et al., 2002 **[27]	Passenger transport companies	316	**C**-**S**	P: 24–70%	15%	> 1	Upper/lower extremities, neck, shoulders, back, hips	Six pain localisations
**Dragano et al., 2003 **[28]		316	**C**-**S**	P: 16–51%	23–64%	Tertiles	Neck, shoulders, back, hips	Four pain localisations
**Rugulies et al., 2007**		1,179	**PC**	I: 25–26%	-	Quartiles, continuous	Lower back, neck	Two pain localisations
**Peter et al., 1998 **[29]		1,325	**C**-**S**	P: 19–59%	42–46%	> 1	General MSDs	Three occupational groups
**Lau, 2008 **[22]	Public authorities	1,803	**C**-**S**	-	5.4%	> 1	Neck, shoulders, back, arms, feet	
**Lapointe et al., 2012**		2,431	**PC**	I: 5.6–11%	27–28%	> 1	Neck, shoulders, lower back, upper extremities	Gender
**Taleb et al., 2005 **[35]	Bank/insurance companies	247	**C**-**S**	P: 34%	-	> 1	Lower back	
**de Jonge et al., 2000 **[30]	Random sample from the general population	11,175	**C**-**S**	P: 11%	-	Tertiles	Upper/lower extremities, nape of neck, shoulders, back	
**Toivanen, 2011 **[31]		2,613	**C**-**S**	P: 22–29%	24–30%	> 1	Upper/lower extremities, shoulders, back	Gender
**Bernard et al., 2011 **[32]	Vineyard workers	3,947	**C**-**S**	P: 21–58%	8–13%	> 1	Upper/lower extremities, nape of neck, shoulders, back	Gender
**Tsutsumi et al., 2001 **[24]	Dental technicians	105	**C**-**S**	-	-	> 1	Upper extremities, nape of neck, shoulders, back	
**Krause et al., 2010 **[38]	Call centre operatives	165	**PC**	I: 52%	3%	> 1, continuous	Nape of neck and shoulders, right and left upper extremities	Three pain localisations
**Burgel et al., 2010 **[33]	Cleaners	439	**C**-**S**	P: 56%	54%	> 1, quartiles, continuous	Shoulders	
**von dem Knesebeck et al., 2005 **[34]	Police officers	480	**C**-**S**	P: 13–50%	19–66%	> 1	Nape of neck, shoulders, back, hips	Four pain localisations

Legend: P= prevalence, I = Incidence, C-S = Cross-sectional study, PC = Prospective cohort study, C-C = Case-control study, NA = not applicable.

The other seven studies which did not incorporate a dichotomous ERI variable (values > 1 vs. values ≤ 1) defined ERI using tertile or quartile limits [21,23,26,28-30,36] or only used the continuous ERI ratio score in their computations [39]. In the studies which stipulated an ERI ratio score greater than 1, the frequency of an effort-reward imbalance was between 3% and 66% (Table 3).

A range of 5.6% to 70% was observed for the prevalence or incidence of musculoskeletal pain. Pain was reported in the following parts of the body: neck, shoulders, upper extremities, back, hips and lower extremities (Table 3). Three studies logged pain in only one part of the body [25,33,35]. The other 16 papers recorded pain in at least two parts of the body. In five of these studies, the pain reported was separately assessed by area [27,28,34,36,38], while the other eleven papers collated pain in different parts of the body within a single variable [21-24,26,29-32,37,39]. The following standardised measures were used for assessing musculoskeletal pain: Nordic questionnaire [44], van Korff chronic pain grade [45], SHC – scoring system for subjective health complaint inventory [46], Berger-Schmitt questionnaire [47], Freiburger Beschwerdeliste [48], Wiholm and Arnetz Questionnaire [49], QuickDASH questionnaire [50] and the Roland-Morris scale [51]. In all other cases, the questionnaires were not further specified.

Furthermore, five studies used stratification based on other criteria: Lapointe et al. [37] examined the gender-specific interaction between ERI and posture in workers at Canadian public authorities with computer-based jobs. Toivanen [31] studied working men and women from a Swedish survey and Bernard et al. [32] completed a gender-specific comparison of French vineyard workers. Peter et al. [29] stratified employees at passenger transport companies by different occupational groups: administrative staff, mechanics/skilled manual workers and drivers. Simon et al. [26] stratified caregivers in seven European countries by type of facility: hospitals, care homes and outpatient care.

All 19 studies could be included in the analysis of the ERI hypothesis: 13 studies (Table 4) showed a positive statistically significant association; ten of these were cross-sectional [21-23,25,26,28,30,31,33,34], two used a cohort design and one was a case–control study [36,37,39]. Six studies found no relationship between ERI and musculoskeletal pain. Of the 14 high quality studies, nine identified an association while five did not. Five studies examined the effect of ERI on workers in the healthcare sector [21,23,25,26,39] and all of these found a positive association for the ERI hypothesis.

**Table 4:** Results of the hypotheses

**Source**	**Population**	**Design**	**ERI risk predictor**	**ERI hypothesis**	**OVC risk predictor**	**OVC hypothesis**	ERI*OVC risk predictor	ERI*OVC hypothesis	**Quality**
**Herin et al., 2011 **[25]	Health care workers	**C**-**S**	+	↑					High
**van Vegchel et al., 2002 **[21]		**C**-**S**	+	↑					Moderate
**Weyers, 2006 **[23]		**C**-**S**	+	↑	-	↓	+	↑	High
**Simon et al., 2008 **[26]		**C**-**S**	+ + +	↑					High
**Gillen et al., 2007 **[39]		**C**-**C**	+	↑					Moderate
**Joksimovic et al., 2002 **[27]	Passenger transport companies	**C**-**S**	- - - + - -	↓	+ - - + - +	↑			High
**Dragano et al., 2003 **[28]		**C**-**S**	- - + +	↑	- + - -	↓			High
**Rugulies et al.,2007**		**PC**	+ +	↑					High
**Peter et al., 1998 **[29]		**C**-**S**	+ - -	↓					Moderate
**Lau, 2008 **[22]	Public authorities	**C**-**S**	+	↑	+	↑	-	↓	High
**Lapointe et al.,2012**		**PC**	+ -	↑			-	↓	High
**Taleb et al., 2005 **[35]	Banks/insurance companies	**C**-**S**	-	↓	+ - - -	↓			High
**de Jonge et al., 2000 **[30]	Random sample from the general population	**C**-**S**	+	↑			-	↓	Moderate
**Toivanen, 2011 **[31]		**C**-**S**	+ +	↑					Moderate
**Bernard et al., 2011 **[32]	Vineyard workers	**C**-**S**	+ + - + - - - -	↓	- + + - - + - -	↓	-	↓	High
**Tsutsumi et al., 2001 **[24]	Dental technicians	**C**-**S**	-	↓	+	↑			High
**Krause et al., 2010 **[38]	Call centre operatives	**PC**	- + -	↓					High
**Burgel et al., 2010 **[33]	Cleaners	**C**-**S**	+	↑					High
**von dem Knesebeck et al., 2005 **[34]	Police officers	**C**-**S**	+ + - +	↑	+ + - -	↑			High

Legend: + = Positive statistically significant association, - = No statistically significant association, ↑= Hypothesis confirmed, ↓= Hypothesis not confirmed, C-S = Cross-sectional study, PC = Prospective cohort study, C-C = Case-control study.

Furthermore, Krause et al. observed negative confounding for physical work-related stress [38]. This means that the association between ERI and musculoskeletal pain is concealed by confounding and only becomes clear after adjustment [52].

Eight cross-sectional studies presented data for reviewing the OVC hypothesis (see Table 4). All of these were high quality. Four studies found an association between OVC and musculoskeletal pain [22,24,27,34]. Lapointe et al. [37] adjusted their analysis for over-commitment, but did not present any risk predictors or level of significance. For this reason, this prospective cohort study could not be taken into account.

Five studies looked into the relationship between ERI and OVC (see Table 4). One high quality cross-sectional study [23] identified an interaction effect, but the other three cross-sectional studies [22,30,32] and one cohort study [37] observed no relationship.

### Evidence

#### ERI hypothesis

Of the 13 studies with a positive association, ten were cross-sectional, of which seven were of good quality (Table 4). The case control study was deemed to be of moderate quality, while the two prospective cohort studies were of good quality. The studies with no positive association did not have a statistically significant result or produced multiple statistically insignificant partial results. On the basis of one case control study and three prospective cohort studies, and excluding the cross-sectional studies, the evidence for the association between ERI and musculoskeletal pain was graded as moderate. As the cohort studies assessed ERI at baseline and adjusted for baseline pain, we examined how the relationship and musculoskeletal pain changed over time.

#### OVC hypothesis

Four good quality cross-sectional studies indicated a positive association between over-commitment and musculoskeletal pain; another four cross-sectional studies found no association (Table 4). The evidence of a relationship between OVC and musculoskeletal pain was therefore considered to be inconclusive.

#### ERI*OVC interaction hypothesis

Five studies examined a potential interaction between ERI and OVC in relation to musculoskeletal pain. Of these, one cross-sectional study found a positive association (Table 4). On this basis, the evidence for this association was considered to be inconclusive.

## Discussion

The objective of this systematic review was to evaluate the level of evidence for the association between the psychosocial factors determined using the ERI model and work-related musculoskeletal pain. On the basis of the small number of relevant original studies, the results can be summarised as follows:

1. The ERI hypothesis that high efforts combined with a low level of rewards increases the risk of musculoskeletal pain occurring was tested in all the studies included. Based on the results of three cohort studies and one case–control study, a moderate level of evidence was identified for the association between ERI and musculoskeletal pain.

2. The OVC hypothesis was tested in eight studies. This proposes that, greater inclination towards over-commitment increases the risk of suffering from musculoskeletal pain, regardless of effort-reward imbalance,. The results of the eight cross-sectional studies show inconclusive evidence of an association between OVC and musculoskeletal pain.

3. The interaction hypothesis was tested in five studies. This hypothesis proposes that workers with a greater inclination towards over-commitment who also experience an effort-reward imbalance are at the greatest risk of developing work-related musculoskeletal pain. Inconclusive evidence for this association was derived on the basis of one cohort study and four cross-sectional studies.

Taking the entire ERI model into account – i.e. if the three hypotheses are viewed as connected components of this work-related stress model – the synthesis shows a low overall level of evidence for the association between psychosocial factors ascertained using the ERI model and work-related musculoskeletal pain.

If the hypotheses are viewed independently of one another, we can see a moderate level of evidence for the ERI hypothesis and inconclusive evidence for the OVC and ERI*OVC hypotheses. It is debatable whether it makes sense to classify the level of evidence for each hypothesis or whether the overall evidence should be defined for the model as a whole.

We believe that this is the first review to examine solely the association between psychosocial factors in the ERI model and work-related musculoskeletal pain. In their reviews, van Vegchel et al. and Tsutsumi et al. [14,17] looked into the relationship between purely psychosocial factors defined in the ERI model and various disorders - on the basis of 45 and 42 studies, respectively.

In these reviews, the studies on musculoskeletal pain are categorised within the group of psychosomatic symptoms [21,24,27-30]. For the latter group, Van Vegchel et al. [14] report an accordance rate of 87% with the ERI hypothesis, i.e. 13 of 15 studies indicate a statistically significant association. In addition to musculoskeletal pain, these include a wide range of other symptoms, so that it is difficult to compare these with the results of our review. The present review incorporates 13 studies additional to the most recently published review [14]. Nevertheless, the systematic literature research found only a few primary publications examining the relationship between psychosocial factors as per the ERI model and musculoskeletal pain. The study population was highly heterogeneous, making it prudent to conduct a systematic review with a best-evidence synthesis.

The small number of primary publications limits the significance of this review. Furthermore, the research is dominated by cross-sectional studies, meaning that it is impossible to specify the timing of cause and effect. One explanation for the limited number of primary publications is that the ERI model is a relatively new model of work-related stress - as it was only published in 1996. It has not been examined to the same extent as – for example – the older demand-control model developed by Karasek [10].

The significance of the few primary publications available is reduced further by the fact that the OVC and ERI*OVC hypotheses were less frequently tested than the ERI hypothesis.

The review is also restricted by the heterogeneity of the studies included. The 19 studies examine nine different industries and only half of them used the original ERI questionnaire. This may bias the effect of the independent variables and makes it harder to compare the studies. The different cut-off points for the ERI variables (tertiles, quartiles, > 1) also potentially result in a classification bias, making it more difficult to compare exposed and non-exposed individuals.

In addition to the differences in exposure, we found differences in the target variables. Some studies recorded up to six different pain localisations, while others logged just one. In studies which recorded pain in a single part of the body, it is impossible to rule out potential effects on other parts of the body. Studies which recorded pain in various parts of the body and collated this within a variable may likewise have overlooked an effect on pain in an individual part of the body.

Eight of the 19 studies conducted adequate controls for confounding. In the prospective study, embedded in an RCT [40], Krause et al. [38] identified negative confounding for physical factors. With this in mind, there could be substantial effects concealed in the ERI variables in those studies which did not control for physical factors. This would affect more than half of the studies included in the review. It is clear that controlling for confounding by physical factors is not standard when ascertaining psychosocial factors, although there has long been evidence that it is necessary [9,18,40].

It may also be desirable to apply the criteria “*use of the original ERI questionnaire*” and “*control for physical factors*” in a more critical manner. All the same, this would not alter the moderate level of evidence for the ERI hypothesis. The findings would still be rated as consistent [15], as the crucial study by Lapointe et al. [37] would still be of good quality as it fails in only one quality criterion (*response rate* ≥ *80*%).

Because of the paucity of studies, the confounders and the different measures of exposure, it is impossible to conclude whether the association between psychosocial factors - as defined in the ERI questionnaire - and musculoskeletal pain is weak or strong.

Studies which examine the relationship between psychosocial factors as per the ERI model and certain other outcomes have found clearer results. Longitudinal studies of cardiovascular diseases show that workers with a high ERI ratio score are at increased risk [12,53-55]. Statistically significant elevated risks have also been identified in connection with depression [56-58].

The relationship between musculoskeletal pain and the psychosocial factors in the demand-control model has been examined more frequently. None of the recently published reviews and meta-analyses on the association between psychosocial factors and musculoskeletal pain in longitudinal studies has recorded effort-reward imbalances [7,8,15]. These analyses identify small yet significant effects for the association between psychosocial factors as per the demand-control model (high job strain, high job demands, low job control) and pain in the back, shoulders and nape of the neck. The fact that all of these studies are prospective makes these results more reliable.

Future studies should aim to evaluate the association between psychosocial factors captured by the ERI model and musculoskeletal pain; more longitudinal studies testing all three hypotheses should be conducted. Furthermore, the original ERI questionnaire and the standard ERI ratio cut-off point 1 should be used to make the results comparable. To conduct precise pain assessment, standardised questionnaires should be applied that provide data on different pain localisations (e.g. Nordic questionnaire). In addition, work-related physical factors must be consistently controlled, as this provides the basis for establishing potential associations.

## Conclusions

The association between the psychosocial factors ascertained using the ERI model and the frequency of musculoskeletal pain cannot be conclusively established on the basis of current published data. Thus, it is unclear whether the ERI questionnaire is the right tool for identifying groups at risk of developing musculoskeletal pain. Future longitudinal studies must use consistent methods of recording and classifying exposure, supported by control for physical confounders. It may then become clear whether an evidence-based recommendation can be made to use the ERI questionnaire to identify work-related risk factors for musculoskeletal pain.

## Abbreviations

C-C: Case–control; COPSOQ: Copenhagen Psychosocial questionnaire; C-S: Cross-sectional; ERI model: Effort-reward imbalance model; I: Incidence; JDC model: Job-demand-control model; MSD: Musculoskeletal disorder; NA: Not applicable; NWI-EO Questionnaire: Nursing work index – Extended Organisation Questionnaire; OVC: Over-commitment; P: Prevalence; PC: Prospective cohort.

## Competing interests

The authors declare that they have no competing interests.

## Authors’ contributions

PK, the first reviewer, carried out literature research, screened the articles, conducted the quality assessment and wrote the manuscript. AS, the second reviewer, screened the articles and conducted the quality assessment. UL made suggestions on the systematic literature search and critically read the manuscript. AN revised the manuscript critically for important intellectual content and gave final approval for the version to be published. All authors read and approved the final manuscript.

## Pre-publication history

The pre-publication history for this paper can be accessed here:

http://www.biomedcentral.com/1471-2458/14/37/prepub
